# Counteracting Cascades Challenge the Heterogeneity—Stability Relationship

**DOI:** 10.1111/ele.70158

**Published:** 2025-08-01

**Authors:** Jordi Sola, Tom P. Fairchild, Matthew J. Perkins, James C. Bull, John N. Griffin

**Affiliations:** ^1^ Department of Biosciences, Wallace Building Swansea University, Singleton Park Swansea UK; ^2^ School of Biological and Marine Sciences University of Plymouth, Drake Circus Plymouth UK

**Keywords:** habitat complexity, habitat structure, marine rocky shore, stress gradient, substrate topography

## Abstract

Spatial environmental heterogeneity is widely assumed to enhance ecological stability by promoting refugia, biodiversity and asynchrony. Yet, we lack field experiments testing this fundamental relationship and its underlying mechanisms in naturally assembled multitrophic systems. To address this gap, we monitored experimental substrates replicating topographic heterogeneity on a rocky shore over 3 years. Contrary to theory, heterogeneity showed no net effect on community stability due to four counteracting pathways. Heterogeneity increased stability by (i) providing refugia that enhanced population stability and (ii) boosting species richness, which promoted asynchrony. At the same time, it decreased stability by (iii) reducing a dominant non‐native species and (iv) suppressing consumers, both of which otherwise stabilised community composition. These opposing processes cancelled out the heterogeneity–stability relationship, highlighting the complex and multi‐causal nature of this relationship. We caution against the assumption that increasing heterogeneity universally enhances stability, particularly in systems with strong consumer interactions and dominant species.

## Graphical abstract

This study challenges the long‐standing assumption that environmental heterogeneity inherently promotes ecological stability, using a 3‐year field experiment on rocky shores. By unveiling four distinct and opposing pathways—two stabilising and two destabilising—it reveals the underlying complexity of the heterogeneity–stability relationship and establishes a new framework for understanding how these forces interact in natural multitrophic systems.

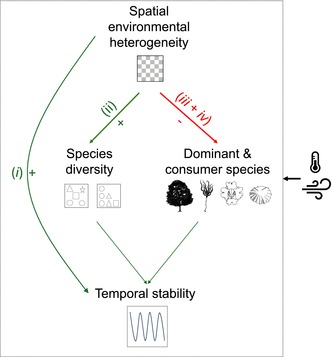

## 1 Introduction

Understanding the drivers of ecological stability is a central concern in modern ecology with far‐reaching implications for sustainability, conservation and restoration (Pinto et al. 2013; Moreno‐Mateos et al. 2020; Wilsey 2021; Li et al. 2021). Temporal stability, the invariability of aggregate community or ecosystem properties through time, captures an ecosystem's capacity to resist internal dynamics and external impacts, as well as to recover from them (Lehman and Tilman 2000; Tilman et al. 2006). A key determinant of temporal stability is the integration of species' diverse and often asynchronous responses to environmental variability and disturbance (Yachi and Loreau 1999; Morin et al. 2014). By shaping the diversity and abundance of species within communities, external factors can potentially exert cascading influences on temporal stability and the long‐term reliable delivery of ecosystem services (Chen et al. 2021; Segrestin et al. 2024).

Multiple pathways contribute to temporal stability, with biodiversity, dominant species and consumers all potentially influencing population stability and promoting asynchronous dynamics that buffer community‐level fluctuations (Tilman et al. 1998; Griffin et al. 2009; Sasaki and Lauenroth 2011; Wagg et al. 2022; Lisner et al. 2024). In turn, environmental heterogeneity has also been broadly linked to stability by ameliorating environmental disturbance and enhancing resistance and resilience (Palmer et al. 1997; Benton et al. 2003; Carey 2003; Brown 2007; Wang and Loreau 2016). This relationship is exemplified by the thermally stable microclimates created by biogenic habitat formers such as oysters and the temperature‐buffering role of rocky habitats, which help mitigate thermal extremes associated with climate change (García et al. 2020; McAfee et al. 2022). Despite its theoretical relevance, the co‐existing mechanisms behind heterogeneity effects on temporal stability—on multiyear temporal scales beyond specific disturbance‐response dynamics (Hurtley 2001; Donohue et al. 2016)—remain poorly tested in real‐world, multitrophic ecosystems, where complex species interactions and environmental gradients may alter expected outcomes.

Heterogeneity is hypothesised to promote temporal stability through multiple mechanisms. First, heterogeneous and complex structures can mitigate disturbance impacts by providing refugia for stress‐sensitive species, enhancing population‐level stability (Oliver et al. 2010). Second, heterogeneity promotes biodiversity by generating varied niches, which in turn allows for more asynchronous species fluctuations and increased stability (Arriero et al. 2006; Bartels and Chen 2010; Wilcox et al. 2017; Kutiel et al. 2023; Mintrone et al. 2024; Lisner et al. 2024). Third, these varied niches may also help reduce competitive exclusion and reduce the prevalence of dominant species and their effects (Sasaki and Lauenroth 2011). Fourth, spatial heterogeneity can also reduce consumer pressure and stabilise population dynamics by creating patchy environments that limit predator movement, moderate predation and provide refuges that promote species coexistence (Huffaker 1958; Murdoch 1977; Hassell 1980; Griffin et al. 2009; Ong et al. 2018). While the few existing empirical studies report a positive heterogeneity–stability relationship (Brown 2007; Qiao et al. 2023), these effects may be offset if heterogeneity suppresses dominant, stabilising species or shelters stress‐sensitive taxa, potentially leading to context‐dependent or even destabilising outcomes.

Rocky shores offer a well‐established model system to test how heterogeneity influences stability through multiple mechanisms. Structural features like crevices and pits create microhabitats that ameliorate desiccation, wave exposure and grazing, providing refugia that enhance the stability of stress‐sensitive species (Hills et al. 1999; Chapman 2000; Kovalenko et al. 2012). This fine‐scale heterogeneity promotes biodiversity by offering diverse niches that can support asynchronous species dynamics (Scrosati and Heaven 2007; Watt and Scrosati 2013). At the same time, increased niche availability can reduce competitive exclusion and dominance by stress‐tolerant species, especially in the mid and low shore where lower environmental stress allows for stronger species interactions (Boaventura, Alexander, et al. 2002; Boaventura, Cancela da Fonseca, et al. 2002; Scrosati, Genne, et al. 2011; Scrosati, Knox, et al. 2011). Furthermore, physical patchiness can disrupt consumer movement and access to prey, dampening top‐down effects (Chapman 2000; Griffin et al. 2009). Finally, the strong environmental stress gradient across shore zones alters species diversity and interaction strengths, offering a natural range of contexts to assess the robustness of heterogeneity–stability relationships (Scrosati, Genne, et al. 2011; Scrosati, Knox, et al. 2011). Together with rapid community development, these features make rocky shores excellent grounds for experimentally testing heterogeneity–stability mechanisms (Hawkins et al. 2020).

In this study, we test the hypothesis that heterogeneity promotes multiyear temporal stability by buffering disturbances and supporting biodiversity. Specifically, we assess whether heterogeneity enhances the temporal stability of intertidal community cover through four pathways: (i) increased population stability via buffered microhabitats, (ii) greater species asynchrony that spreads risk and dampens effects of individual species' declines, (iii) reduced dominance effects that boost richness and asynchrony, but may weaken dominant‐species stability and (iv) reduced top‐down control that stabilises trophic interactions, but may favour stress‐sensitive species. We conducted a 3‐year field experiment along an intertidal gradient using paired artificial substrates with contrasting substrate heterogeneity (flat vs. pitted; Fairchild et al. 2024). Seasonal community surveys and structural equation modelling were used to assess how heterogeneity shaped community structure, population dynamics and multi‐year stability. Our results reveal both stabilising and destabilising effects, challenging theoretical expectations and underscoring the complexity of heterogeneity–stability relationships in multitrophic systems.

## 2 Methods

### 2.1 Study Site & Experimental Design

**FIGURE 1:**
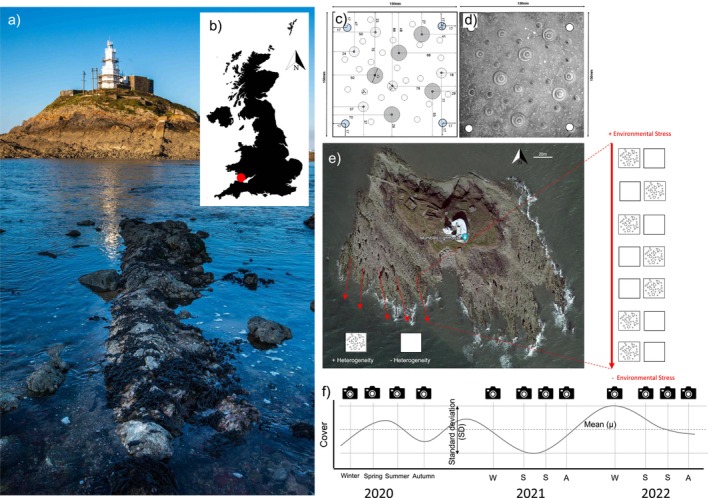
Overview of the experimental set‐up testing the effects of heterogeneity on temporal cover stability. In an intertidal experimental site at Bracelet Bay, Swansea, Wales, United Kingdom (a, b), heterogeneous tiles (c, d), with a standardised set of pits of various sizes and configuration, were deployed alongside non‐heterogeneous controls. The experiment was deployed across 35 stations with two tiles each (one heterogeneous, one homogeneous) along five transects on the shore (e), where each transect ran from the high shore (high emersion stress) to the low shore (low emersion stress). Within each transect, seven stations consisting of a tile representing the heterogeneous treatment (as per figures c and d) and a smooth tile representing the non‐heterogeneous treatment were established, with tiles randomly positioned within a station. Tiles were sampled 11 times during the study period (f).

Between May 2019 and April 2022 (35 months), an experiment was conducted on a moderately exposed rocky shore at Bracelet Bay, Swansea, United Kingdom (51.566, −3.971; Figure 1). The site, within a macro‐tidal, semidiurnal regime (spring tidal range ~8.5 m; neap ~4 m), features a steep upper shore with barnacle‐dominated channels; a mid‐shore with a mix of macroalgae, barnacles, mussels and molluscan grazers; and a gently sloping lower shore dominated by macroalgae together with grazers. Along this emersion stress gradient—from high desiccation stress in the upper shore to reduced stress in the lower shore—35 pairs of experimental limestone tiles representing two heterogeneity levels were deployed on exposed rock across 35 stations (i.e., each station being smaller than 3 m^2^) along five transects, which were positioned haphazardly within 20 m of one another to minimise wave exposure variability. To prevent canopy algae from altering tile conditions (e.g., via temperature buffering, wave attenuation or whip‐lash disturbance; Petrowski et al. 2016), surrounding fucoids and large red algae were regularly cleared. Tiles were sampled seasonally during low tide, yielding 11 time points per tile.

Experimental heterogeneous tiles (15 × 15 cm) were created by drilling a set of large, medium and small pits following a standardised configuration, mimicking small‐scale depressions formed by weathering and bio‐erosion (Kázmér and Taborosi 2012; Naylor et al. 2012), and providing heterogeneity in surface texture and moisture retention. Meanwhile, non‐heterogeneous tiles consisted of flat tiles without any pits (see Fairchild et al. 2024 for a detailed account on the experimental tiles). Importantly, as the additional surface area created from the pits was outweighed by random variability in tile sizes that resulted from manual cutting of the rock into tiles, realised surface area was indistinguishable between non‐heterogeneous and heterogeneous tiles (Fairchild et al. 2024).

Modifications of substrate heterogeneity have been widely used on many natural and artificial substrates on rocky reefs to alter local conditions including temperature, moisture, wave disturbance and grazing (e.g., Airoldi and Cinelli 1997; Bulleri 2005; Hawkins et al. 2020) and are generally linked to the variability in environmental conditions (e.g., Stein et al. 2014; Agra et al. 2023; Sola and Griffin 2025). The single form of heterogeneity applied allows high replication within heterogeneity levels, which is necessary to decipher complex cascading effects within a noisy natural system. The design thus provides a case study of one form of heterogeneity that is widely found on rocky shores.

### 2.2 Data Collection

Tiles were sampled using image analysis following stratified sampling—taking pictures of the canopy and understorey strata separately (Supporting Information S2 for a more detailed account on data collection). Understorey species cover was estimated using point‐count image subsampling, in which all organisms located directly beneath each of 500 grid points were identified. In contrast, canopy species cover was measured using image analysis in Photoshop. We accounted for ‘edge effects’ by excluding a 1.7‐cm area around the edge of the tile from image analysis. We also corrected for the bias from the presence of bolts fixing the tile to the substrate, which would have created topography that benefited organisms settling on the tile (more details in Supporting Information S2). Taxonomic identification was to the lowest level possible. Lastly, we estimated emersion ratio (the proportion of time a tile is tidally exposed/emersed over a year) as a continuous variable by combining directly measured emersion times with local water level data (Supporting Information S2). Using this continuous variable, we obtained a categorical variable to facilitate comparisons across zones by grouping stations into high, mid and low shore ensuring that the same number of tiles/stations was included in each group.

### 2.3 Heterogeneity Effects on Community Diversity and Composition

We tested for heterogeneity effects on community diversity and composition and their interactive effects with the emersion gradient. To do so, we calculated the average of each pertinent diversity and cover metric over time for each individual tile across the study period. Using mixed effects linear models (Bates et al. 2009), we tested the effects of heterogeneity, emersion ratio and their interaction on species richness and species evenness (Pielou Index). Fixed factors were z‐transformed so that all variables were on a similar scale. We also included the random effects of transect and station nested within transect. Model diagnostics, including normality and heteroscedasticity of residuals for all linear models, were checked visually (Hartig and Hartig 2017). When needed, data were log‐transformed or square‐rooted to meet assumptions.

Using the same modelling approach, we tested for heterogeneity effects on the most abundant or known strongly interacting species or species groups, since dominant and strongly interacting species are known to influence temporal stability (Sasaki and Lauenroth 2011). The invasive barnacle *Austrominius modestus* was considered separately due to its dominant and seasonal occurrence, while native barnacles such as *Semibalanus balanoides* , *Chthamalus montagui* , *Balanus* spp. and *Perforatus perforatus* were grouped together. Macroinvertebrate consumers, largely represented by *Patella* spp. and *Nucella lapillus*, were grouped. Both grazers and predators have been shown to contribute to cascading influences (Silliman et al. 2013) and limpets and whelks both reduce densities of barnacles and mussels (Berlow and Navarrete 1997; Hunt and Scheibling 1998), but with *Nucella* presenting patchy occurrence and a minor role in the community (Supporting Information S7). The opportunistic and ephemeral seaweeds were also grouped as *Ulva* spp., *Cladophora* sp. and *Porphyra dioica* as found in previous surveys (Knoop et al. 2020). Other suspension feeders were initially considered as a group (e.g., *Mytilus edulis* , *Sabellaria alveolata* , *Pomatoceros* spp.). However, after including them in the SEM we found no relevant links with stability and since they responded to heterogeneity and emersion following the same patterns as species diversity, we excluded them resolving that the effects of heterogeneity on them were already captured within species diversity responses. We used mean consumer cover, which was representative of cover within any single time point (Pearson's *r* = 0.75; Supporting Information S3).

### 2.4 Heterogeneity Effects on Temporal Stability and Stability Mechanisms

We quantified five ecological stability‐related metrics including temporal stability and underlying mechanisms for the whole community (Supporting Information S4). We assessed aggregate temporal stability using the inverse of the coefficient of variation (1/CV) of total cover, calculated per tile based on its temporal mean and standard deviation (Tilman 1996). In addition, we computed complementary metrics—compositional stability, population stability, species asynchrony and statistical averaging—using time‐series data per tile. Although biomass or biomass production is commonly used in stability studies in other systems, following previous rocky shore studies (Bulleri et al. 2012; Valdivia et al. 2013; Mintrone et al. 2024) we chose total cover as it: (i) directly measures the space occupied by species, a limiting resource on rocky shores (Connolly and Muko 2003); (ii) facilitates a unified measure across diverse species with contrasting traits; and (iii) unlike biomass, it is a nondestructive descriptor suitable for repeated sampling in situ (e.g., Valencia et al. 2020). Temporal stability in aggregate properties such as total biomass or cover effectively captures whole community‐level responses to environmental variation, integrating across population fluctuations of individual species (Loreau et al. 2002; Gonzalez and Loreau 2009; Wisnoski et al. 2023; Kolasa et al. 2024).

We calculated temporal stability after detrending to remove a weak long‐term trend in mean cover, isolating temporal fluctuations from broader directional temporal changes (Craven et al. 2018; Supporting Information S5). Communities established quickly, with diverse species present after the first recruitment season, and analyses confirmed that the first year did not disproportionately influence temporal stability (Supporting Information S5). As a result, all time points were included in the analysis. We then assessed how environmental heterogeneity along the emersion gradient influenced temporal stability, along with the underlying mechanisms of population stability, species asynchrony, statistical averaging and compositional stability, using mixed‐effects linear models aligned with those used to assess community components (Baselga et al. 2018).

### 2.5 Pathways Explaining Heterogeneity Effects on Temporal Stability

We built a multigroup piecewise structural equation model (pSEM; Lefcheck 2016) to identify and quantify the pathways linking environmental heterogeneity to temporal stability. A hypothetical causal model was first developed based on a priori theory and ecological knowledge of rocky shore communities (Supporting Information S6), including direct paths from heterogeneity to temporal stability and indirect paths via species groups and stability mechanisms. The multigroup analysis allowed us to assess how these pathways varied along the emersion gradient, categorised into low, mid and high shore zones. All component models within the pSEM were fit using mixed‐effects linear models, following the approach previously described for models assessing spatial differences across community components.

Partial bivariate correlations were included based on theoretical expectations or when directed‐separation (d‐separation) tests indicated significant associations between variables not linked in the initial model. D‐separation tests evaluate whether the model implies conditional independence between variable pairs, with significant results suggesting a missing path or correlation. For the overall multigroup model, a Fisher's *C* test—a global goodness‐of‐fit measure—was used to assess whether any relationships implied by the data were missing from the model structure. To facilitate model convergence and reduce degrees of freedom, mixed‐effect models were simplified to linear models when this did not substantially alter coefficient estimates or the direction of effects. Finally, to quantify the cascading effects of heterogeneity on stability metrics, we calculated the product of significant path coefficients along each causal chain and summed these to estimate the overall effect of heterogeneity on temporal stability and its underlying stability mechanisms.

## 3 Results

### 3.1 Heterogeneity Effects on Community Diversity and Composition

**FIGURE 2:**
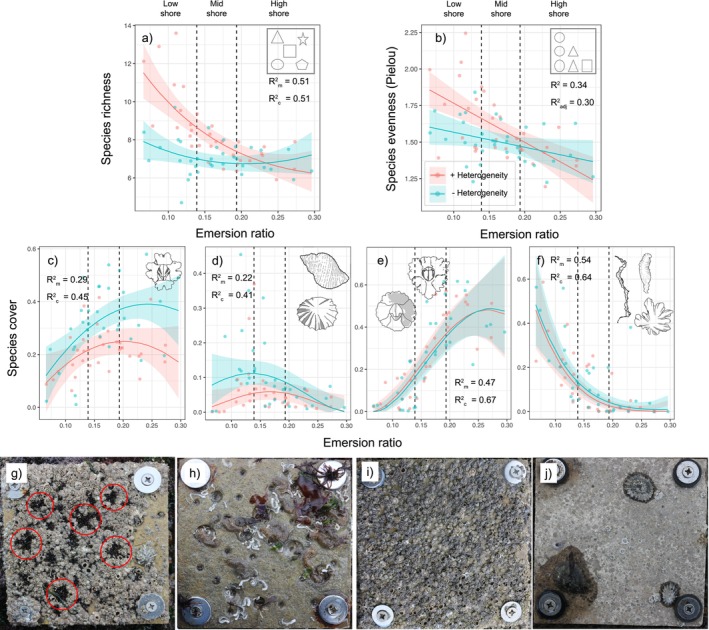
Heterogeneity effects on species diversity and the abundance of species groups along the emersion gradient in rocky shore communities. (a–f) Linear model results (mean ± 95% CI) for the effects of emersion ratio and heterogeneity on: (a) species richness, (b) Pielou evenness, (c) *Austrominius modestus*, (d) macroinvertebrate consumers, (e) native barnacles and (f) ephemeral macroalgae. Lines represent predicted responses from mixed‐effects models (via *ggpredict* in *R*); points are per‐tile means across sampling dates (*n* = 67). Marginal and conditional *R* ^2^ values (*R* ^2^m and *R* ^2^c) are shown within each graph (g–j). Observed field patterns: In the high shore (g), mussels, snails and barnacles survived in pits (red circles) but not on smooth tiles; in the low shore (h), pits supported higher recruitment and survival, increasing diversity and altering composition. High‐shore smooth tiles were often dominated by *A. modestus* (i), while consumers (e.g., *Patella* spp.) were more common on mid‐ and low‐shore tiles, reducing cover through grazing (j).

Heterogeneity increased biodiversity in terms of both species richness (*t*_het × emersion_ = 3.74, *p* < 0.001) and species evenness (*t*_het × emersion_ = 2.15, *p* = 0.03) in the low shore, with effects gradually decreasing until disappearing in the high shore (Figure 1a,b). The increase in biodiversity was tightly linked to the presence of pits, as mussels, snails and other invertebrate species proliferated inside the pits in the mid and high shore, while polychaetes and non‐ephemeral macroalgae grew inside the pits in the low shore (Figure 2g,h).

Dominant species and other species groups, however, showed differential responses to heterogeneity and the emersion stress gradient. Heterogeneity reduced the cover of the dominant non‐native barnacle *Austrominius modestus* in the mid and high shore (*t*_het × emersion_ = 2.26, *p* = 0.030; Figures 2c and 1i), and the cover of macroinvertebrate consumers (*t*_het_ = 2.42, *p* = 0.020; *t*_emersion_ = 2.13, *p* = 0.039; Figures 2d and 1j). No heterogeneity effects were recorded for native barnacles (*t*_het_ = −0.90, *p* = 0.37; Figure 2e) or ephemeral macroalgae (*t*_het_ = 0.05, *p* = 0.957; Figure 2f), which showed sharp positive and negative responses to emersion ratio, respectively.

### 3.2 Heterogeneity Effects on Temporal Stability and Stability Mechanisms

**FIGURE 3:**
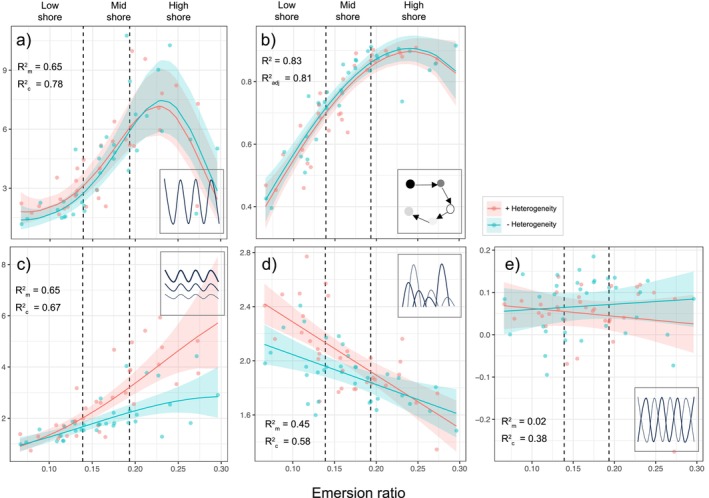
Heterogeneity effects on temporal stability and stability mechanisms along the emersion gradient. Results from linear models presented as original data and linear model estimates (mean ±95% CI) for (a) temporal stability, (b) compositional stability, (c) population stability, (d) statistical averaging and (e) species asynchrony along the intertidal emersion gradient. Points in each graph are the mean of all observations across dates per tile (*n* = 67). Marginal and conditional *R* ^2^ values (*R* ^2^m and *R* ^2^c) are shown within each graph. Lines are the predicted response obtained from the mixed‐effects models used to test emersion ratio and heterogeneity effects using the *ggpredict* function in *R*.

No net heterogeneity effects were recorded for temporal stability (i.e., 1/CV, see Section 2: Methods; *t*_het_ = −1.69, *p* = 0.100; Figure 3a) or compositional stability (i.e., movement in multivariate community space, see Supporting Information S4; *t*_het_ = 0.50, *p* = 0.616; *t*_emersion_ = 1.51, *p* = 0.135; Figure 3b) along the emersion gradient. Instead, temporal stability strongly and non‐linearly responded to emersion stress, peaking in the lower part of the high shore, but declining rapidly as emersion stress increased further. In contrast, heterogeneity increased population stability in the high shore (*t*_het × emersion_ = −2.53, *p* = 0.015; Figure 3c) and increased statistical averaging at low and mid shore levels (*t*_het × emersion_ = 2.11, *p* = 0.042; Figure 3d). Finally, no heterogeneity effects were found on species asynchrony (*t*_het_ = −0.71, *p* = 0.477; *t*_emersion_ = −0.80, *p* = 0.422; Figure 3e).

### 3.3 Pathways Explaining Heterogeneity Effects on Temporal Stability

Four countervailing pathways stemming from heterogeneity helped explain the absence of net heterogeneity effects on temporal stability and were mediated by (1) population stability, (2) species asynchrony, (3) a dominant species *Austrominius modestus* and (4) consumers (Figure 3a–h). In addition, a key link (i.e., heterogeneity effects on species richness) varied across shore levels, which affected two of the four pathways (i.e., pathways #1 and #2). The other two pathways did not vary across the shore (Supporting Information S1).

**FIGURE 4:**
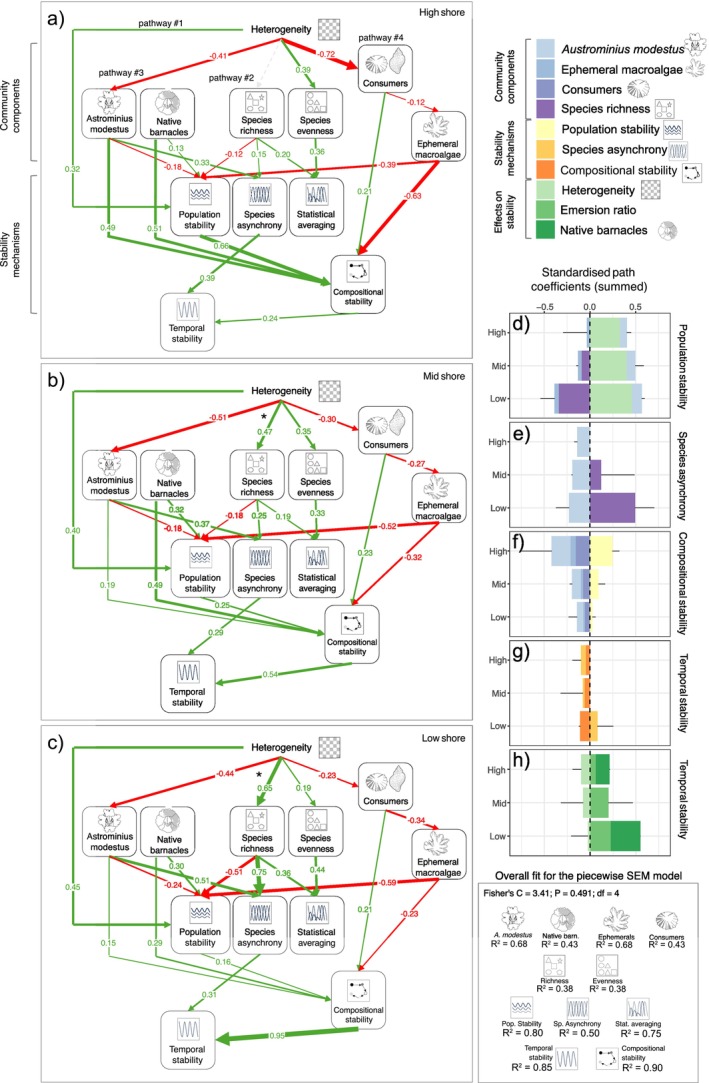
Heterogeneity effects on temporal stability and underlying mechanisms across the shore. Left panels (a–c) show multigroup piecewise SEMs illustrating how heterogeneity influenced temporal stability via community components (e.g., species richness) and stability mechanisms (e.g., species asynchrony). Green, red and grey lines indicate significant positive, significant negative and non‐significant paths (*p* > 0.05) that changed across shore heights; numbers are standardised path coefficients (SPCs). Black asterisks denote significant differences across shore levels (see Supporting Information S1). SEM fit metrics (Fisher's C, *R* ^2^) are shown in the lower right corner. Right panels (d–h) summarise SPCs for heterogeneity effects on population stability, species asynchrony, compositional stability, temporal stability and net effects (including emersion gradient and native barnacles). Error bars reflect cumulative uncertainty across all component coefficients per case, which are not generalisable to individual effects. Notably, indirect pathways include all links originating from heterogeneity and cascaded through community components (e.g., consumers, species richness) and stability mechanisms (e.g., species asynchrony) down to temporal stability.

Consistent with expectations, heterogeneity directly promoted population stability (pathway #1; Figure 4d). In the low shore, however, these direct positive effects were largely counteracted by indirect negative effects on population stability mediated by consumers and species richness. Nevertheless, overall, the population stability pathways helped promote compositional stability (Figure 4f) and ultimately temporal stability along the emersion gradient, particularly in the mid and high shore (Figure 4g). Also in line with expectations, heterogeneity promoted asynchrony, and in turn temporal stability, by increasing species richness (pathway #2; Figure 4e). Notably, the influence of heterogeneity on species richness was the only link to vary significantly between emersion zones, with no detectable effect in the high zone and increasingly positive effects in the mid and low zones (Figure 4a–c).

Yet, unexpectedly, heterogeneity reduced temporal stability through species asynchrony and compositional stability by reducing the cover of a dominant and largely stabilising non‐native barnacle, *Austrominius modestus* (pathway #3; Figure 4e,f). The decrease in *A. modestus* cover counteracted positive effects of heterogeneity on population stability (mechanism #1; Figure 4f) and species richness (pathways #2; Figure 4e). Furthermore, heterogeneity decreased the effects of stabilising consumers (pathway #4; Figure 4a–c). Reduced consumer effects directly diminished compositional stability (Figure 4f). Indirectly, reduced consumer effects reduced both compositional stability and population stability by decreasing their negative effects on ephemeral macroalgae (Figure 4d,f). Collectively, decreased consumer effects reduced temporal stability, particularly through reduced compositional stability.

Besides heterogeneity, emersion ratio (even within low, mid and high zones) and the abundance of native barnacle species—unaffected by heterogeneity—had larger effects on temporal stability (Figure 4h). The combination of these two additional drivers, together with the counteracting pathways described above, contributed to further obscure net heterogeneity effects along the emersion gradient (Figure 3a).

## 4 Discussion

**FIGURE 5:**
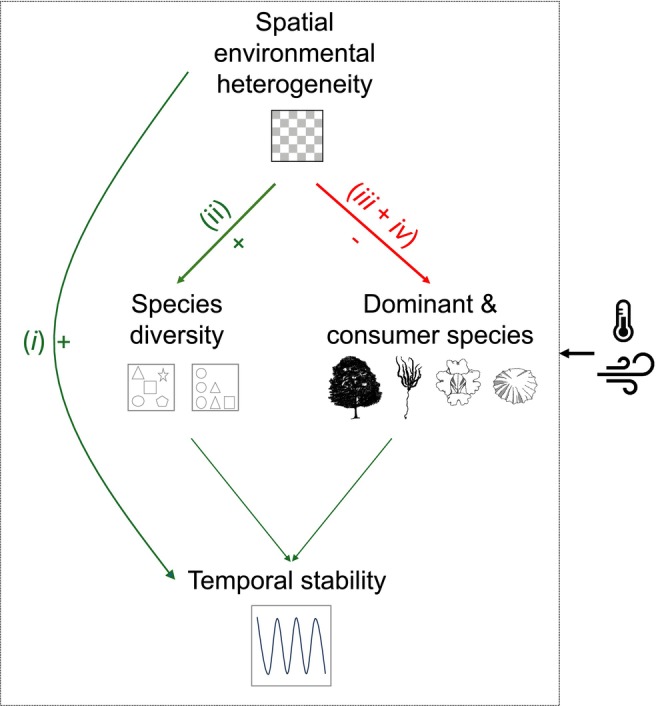
Heterogeneity influences temporal stability through multiple counteracting cascades. Heterogeneity effects on temporal stability were negated due to counteracting positive and negative pathways, which consisted of (i) direct stabilising refugia effects (population stability), (ii) diversity‐mediated stabilising effects (asynchrony) and destabilising effects mediated by suppression of (iii) dominant and (iv) consumer species. Note that effects of heterogeneity via dominant and consumer species might be inherently context‐dependent, hinging on how these important groups respond to heterogeneity and in turn influence stability.

Unravelling the drivers of ecological stability remains a significant research challenge, especially in natural settings where multiple factors interact. Our multiyear experiment on a rocky shore challenges the assumption that environmental heterogeneity promotes temporal stability. Although heterogeneity increased population stability and biodiversity, it did not yield a net positive effect on temporal stability. Structural equation modelling clarified this paradox, supporting that heterogeneity both promoted and undermined stability through various pathways (Figure 5).

Our findings suggest that heterogeneity can enhance stability directly by providing refugia and indirectly increasing species richness (pathways i and ii in Figure 5). By offering varied conditions, including refugia from stressors like desiccation, wave forces and consumers, heterogeneous substrates likely reduced the impact of disturbances and aided population recovery. This aligns with the established role of refugia in helping species survive stress and disturbance (Oliver et al. 2010; Keppel et al. 2012; Selwood et al. 2015). Additionally, heterogeneity enhanced richness by creating new environments where less tolerant species, such as mussels in these semi‐exposed communities, could escape the harsh conditions typical of barnacle‐dominated communities (Kawai and Tokeshi 2007; Barbosa et al. 2022) and by supporting non‐ephemeral macroalgae (e.g., *Fucus* spp.; Jonsson et al. 2006), which would otherwise be removed by storms and consumers. Increased richness, in turn, likely promoted asynchrony through differential responses to environmental variation or density‐dependent competition (Firkowski et al. 2022). This mechanism has been demonstrated at multiple spatial scales in marine environments, where heterogeneity increased asynchrony within and between populations, ultimately enhancing diversity and stability across marine protected areas, and buffering them against marine heatwaves and climate change (Benedetti‐Cecchi et al. 2024).

However, these stabilising effects were counterbalanced by other pathways. Contrary to traditional expectations, the positive effects of heterogeneity were offset by its reduction of a stabilising non‐native dominant barnacle, *Austrominius modestus*, and consumers (pathways iii and iv in Figure 5). The non‐native barnacle likely enhanced stability due to its stress‐ and disturbance‐tolerant traits and its competitive interactions with native species. Its strong influence likely stemmed from its dominance and traits rather than its non‐native status, though non‐natives may in some contexts show greater stability under environmental stress (Buckley and Catford 2016; Gu et al. 2023). Meanwhile, heterogeneity's inhibition of consumers, consistent with previous studies on rocky shores, likely resulted from restricted movement and prey access (Bazterrica et al. 2007; Johnson et al. 2008; Griffin et al. 2009). Since consumers act as a strong ecological filter by eliminating less stress‐resistant, more variable species like ephemeral macroalgae, their reduction destabilised community composition over time (i.e., particularly limpets; see Supporting Information S8). Thus, heterogeneity can inadvertently favour the establishment of more vulnerable species while inhibiting stabilising forces, leading to destabilisation even as it enhances diversity and asynchrony. Many other systems similarly host dominant species, as well as strongly interacting consumers, which might also mediate destabilising effects of heterogeneity on stability.

Our results place the effects of heterogeneity on stability within a broader context of community and environmental drivers. While heterogeneity influenced species richness, population stability and asynchrony, the strongest effects on overall stability occurred along the environmental stress gradient, with stability peaking in areas dominated by stress‐resistant barnacle species. This pattern is consistent with extensive theory and empirical work demonstrating that environmental stress strongly structures species interactions and community composition, particularly in rocky intertidal systems (Grime 1977; Menge and Sutherland 1987; Bertness and Leonard 1997; Bertness and Ewanchuk 2002; Harley and Helmuth 2003; Voß and Schäfer 2017). Although the Stress Gradient Hypothesis predicts that facilitative interactions should increase under harsher conditions (Bruno et al. 2003; Maestre et al. 2009), we found no evidence that heterogeneity‐driven amelioration was stronger in the high shore. Instead, heterogeneity's net effects remained muted across the stress gradient as a result—as discussed above—of countervailing pathways.

Our findings emphasise the need for further exploration and re‐evaluation of heterogeneity–stability relationships. While much effort has been devoted to understanding biodiversity–stability and heterogeneity–diversity relationships (Pennekamp et al. 2018; Agra et al. 2023), far less attention has been given to linking environmental heterogeneity to stability. This is critical, as heterogeneity is often manipulated in applied settings, from nature‐sensitive engineering to ecological restoration, where stability is a desirable outcome to ensure reliable ecosystem service provision (Firth et al. 2024; Silliman et al. 2024). Our novel experimental results imply that enhancing heterogeneity in such settings may not have assumed benefits for ecological stability and, by enhancing biodiversity while potentially suppressing stability, may even drive trade‐offs between biodiversity and stability.

Although our experiment was conducted at a fine spatial scale, this matched the scale at which small‐bodied, low‐mobility organisms interact with their environment through competition, predation and resource use (Price 1983). In contrast, larger bodied organisms, such as birds or mammals, perceive and respond to heterogeneity at coarser spatial scales, as movement generally scales with body size across species (Hillaert et al. 2018; Anderson and Fahimipour 2021; Straus et al. 2022). Thus, while absolute scales vary, aligning experimental scale with the scale of ecological interactions ensures broader relevance of our results. Moreover, fine‐scale heterogeneity can influence community structure and stability at larger spatial scales, as microhabitat processes often scale up to shape landscape‐level dynamics (Puerta‐Pinero et al. 2007; Martirosyan et al. 2013; Choi et al. 2021; With 2019). Therefore, and provided that ecological context and scale are appropriately considered, our mechanistic insights should prove relevant to other systems.

Nevertheless, it must be noted that, while the specific form of rocky shore heterogeneity tested here provides a novel blueprint, it does not capture the full range of possible effects across ecosystems, forms of heterogeneity and spatial scales (e.g., Mintrone et al. 2024). Indeed, the effects of heterogeneity are likely to vary substantially with the form of heterogeneity and the traits of both dominant and consumer species, which may determine context‐dependent effects (Dolezal et al. 2024). Furthermore, although communities assembled rapidly in our study system, compositional changes during succession may influence the relative importance of pathways supporting or undermining stability (Meng et al. 2023; Supporting Information S5). Additionally, while our study assesses effects on compositional and temporal stability, future research should explore other dimensions of stability, such as resistance and resilience, which may respond differently (Donohue et al. 2013). Most importantly, we encourage subsequent studies to embrace the complexity of contemporary ecosystems, including the roles of non‐native species and consumers under different climate change scenarios, to more fully understand heterogeneity–stability relationships in real‐world settings.

## 5 Conclusions

This study reveals the complex, multicausal nature of the relationship between environmental heterogeneity and temporal stability. Although heterogeneity can enhance stability through mechanisms such as population stability and increased biodiversity, these effects can be neutralised by the reduction in dominant and consumer species effects, resulting in no net gain in stability. These findings deepen our understanding of ecological stability and highlight the importance of considering multiple, potentially counteracting pathways in both ecological theory and practice.

## Author Contributions

J.S. and J.N.G. conceptualised the research. J.S., T.P.F. and M.J.P. conducted fieldwork. J.S. collected and analysed the data. J.S. and J.N.G. wrote the manuscript. All authors contributed to the corrections of the manuscript.

## Conflicts of Interest

The authors declare no conflicts of interest.

## Peer Review

The peer review history for this article is available at https://www.webofscience.com/api/gateway/wos/peer‐review/10.1111/ele.70158.

## Supporting information


Data S1.

## Data Availability

Data and R code used for this study are available at https://github.com/JSolaC/Solaetal2025‐Stability. The data are also available at Dryad (DOI: 10.5061/dryad.kprr4xhhg).
