# Supplementary material for: Counteracting Cascades Challenge the Heterogeneity—Stability Relationship
Source: Ecol Lett. 2025 Aug 1;28(8):e70158. doi: 10.1111/ele.70158 (PMC12315071; doi:10.1111/ele.70158)
Supplement: Supplementary file 1 — Data S1. [file ELE-28-0-s001.docx]

**Appendix S1: Counteracting cascades challenge the heterogeneity – stability relationship**

Authors: Jordi Sola^1^, Tom P Fairchild^1^, Matthew J Perkins^2^, James C Bull^1^, John N Griffin^1*^

Addresses:

^1^Department of Biosciences, Wallace Building, Swansea University, Singleton Park, Swansea, SA2 8PP, United Kingdom;

^2^School of Biological and Marine Sciences, University of Plymouth, Drake Circus, Plymouth, PL4 8AA, United Kingdom;

* Corresponding authors: [j.n.griffin@swansea.ac.uk](mailto:j.n.griffin@swansea.ac.uk)

**Table of contents**

1. Structural Equation Modelling (SEM) Multigroup test output
2. Detailed methods on data collection
3. Consumer occurrence
4. Stability metrics used in the study
5. Sensitivity tests regarding the effect of time on community components and temporal stability
6. Metamodel for the testing of heterogeneity effects on Temporal Stability
7. Sensitivity Tests for the Randomised Block Design
8. Sensitivity Tests for the multigroup piecewise SEM model

Supporting Information S1: Structural Equation Modelling (SEM) Multigroup test output

This supplement presents the results of the multigroup *pSEM* analysis, which tested how relationships between variables varied across different levels of emersion stress (high, mid, and low shore zones). The output is summarized in two parts: a) SEM paths that were significantly different from one another across high, mid and low shore, and b) SEM paths that were significantly different from 0 only in some of the three levels of emersion stress.

In Table S1, we list response–predictor pairs where the strength of the causal relationship (i.e., path coefficient) significantly differed among stress levels. These differences were tested using the multigroup functionality in *pSEM*, which allows for the statistical comparison of path coefficients across predefined groups. A significant p-value indicates that the magnitude of a given path varied across at least two emersion zones.

In Table S2, we report cases where a given path was statistically significant in some stress levels but not in others. For each response–predictor pair, we provide the p-value of the path coefficient within each stress level group. This highlights whether a particular relationship was only present (i.e., significantly different from zero) in specific environmental contexts.

Together, these results illustrate that only a few variables varied both in strength and significance across the three emersion stress groups. This indicates that, for the most part, the effects of heterogeneity on temporal stability were consistent across the shore despite the observed variation in community and stability components. However, notably, Table S2 shows that the link Heterogeneity-Species richness varied across the shore, which in turned underpinned mechanisms #1 and #2 mediated by population stability and species asynchrony, respectively.

**Table S1. Differences in multigroup *pSEM* mediator effects across emersion stress levels.** Response and Predictor variables for the significantly different links across any of the emersion rate groups (high stress, mid stress and low stress).

| Response | Predictor | p-value |
| --- | --- | --- |
| Temporal statistical averaging | Heterogeneity | 0.032 |
| Species richness | Heterogeneity | 0.001 |
| Consumer | Emersion rate | <0.001 |
| Native barnacles | Emersion rate | <0.001 |

**Table S2. Differences in the significance of multigroup *pSEM* mediator effects across emersion stress levels.** Response and Predictor variables for the links showing differences in their significance from zero across the emersion rate groups (high, mid and low stress).

| Response | Predictor | High stress | Mid stress | Low stress |
| --- | --- | --- | --- | --- |
| Native barnacles | Emersion rate | 0.575 | <0.001 | 0.161 |
| Consumers | Emersion rate | 0.328 | 0.032 | 0.005 |
| Species richness | Heterogeneity | 0.22 | 0.015 | 0.003 |

Supporting Information S2: Detailed methods on data collection

Tiles were sampled using stratified image analysis – separating the canopy and understorey strata. The canopy stratum was pictured when arriving to the site without moving any organism. Multiple pictures were taken of the understorey after carefully moving the canopy to the sides to uncover the organisms underneath. Images without canopy were trimmed to only include organisms falling within the 126 x 126 mm central area of the tile and avoid edge effects. Point Count Estimation (500 points) subsampling was used for understorey sessile organisms within this area, identifying all organisms falling underneath each point. This avoided underestimating cover and diversity due to the exclusion of overlapping organisms in each point. The percentage cover of rare sessile species (<1% cover), mobile species and canopy was quantified directly (Photoshop 2020), as they could not be properly sampled with Point Count Estimation. All organisms found were identified to the lowest taxonomic level possible, and a conservative approach was applied where image resolution could limit identification accuracy. Three tiles did not present enough time points to be included in the study, reducing the sample size from 70 to 67, as these where very low in the shore and hard to access.

We applied three corrections to the Point Count Estimation dataset. First, we prevented ‘edge effects’ by excluding from image analysis a 3.4 cm area around the edge of the tile. Second, we corrected for proximity to corners, where the four bolts fixing the tiles to the ground created topography that could be confounded with heterogeneity. To do so, we divided the tile image into four equal square corners, and delimited the area of influence to the four rows of Point Count Estimation points closest to each bolt. Using binomial models, we tested whether proximity to bolt changed tile organism occurrence in each square corner, respectively. We repeated the test after sequentially removing each delimited row of Point Count Estimation points until the binomial test was non-significant. Third, we corrected for missing Point Count Estimation points inside pits in heterogeneous tiles due to canopy cover. For instance, heterogeneity effects are based on the identification of organisms in 120 points, resulting in 24% of the Point Count Estimation points falling within the pits. If this percentage fluctuated due to missing points, heterogeneity effects would be over- or underestimated. To correct for this bias, we calculated the ratio of points within and outside pits for each heterogeneous tile image, and applied a weighting factor obtained by dividing the obtained ratio by the expected ratio for both points within and outside pits.

**Figure S1. Corrections applied to Point Counting Estimate data.** Two images of the same tile at different times, illustrating the three applied corrections. First, we corrected for ‘edge effects’ by excluding the outer 3.4 cm wide area around each tile, shown as the grey frame around both images. Second, each image was divided into four corners (blue lines in Panel a), and binomial models were run for total cover and the cover of the five most abundant species groups (barnacles, mussels, ephemeral macroalgae, Fucus spp., and Patella spp.) against the ‘distance to corner.’ If significant, points closest to the corner (red points in Panel a; corner coloured in black) were sequentially removed until the model was no longer significant or up to four points were excluded. Third, we accounted for points falling within pits on the tile (green areas in Panel b) and points outside the pits. If a point was covered and species identification was not possible (e.g., due to coverage by Porphyra spp. or Fucus spp.), it was left blank. For example, in Panel a, several points where Porphyra spp. and Fucus spp. prevented species identification were summed, resulting in 115 points within pits and 350 points outside. These were compared against the total number of points within (120) and outside (364), after excluding 16 points over the bolts in each corner. A correction factor was then applied so that points within pits were adjusted by a factor of 1*(120/115) and points outside pits by 1*(364/350). This ensured that the ratio of points within and outside pits was consistent across all images, preventing over- or underestimation of heterogeneity effects. Note that in both panels, the coloured cells are for illustration purposes and do not perfectly align with the actual points used in the sampling, which are marked by the intersections of the grid lines.

Annual percentage emersion time or emersion ratio (time_emersed_/time_total_) was obtained from the amount of time tiles were emersed during a tidal cycle (i.e., emersion time) and local water height data from the British Oceanographic Data Centre (BODC). First, emersion time per tile was obtained by deploying waterproof temperature loggers (HOBO MX2203) for 24 hours on half the stations, which were carefully selected to consider high, mid and low shore sections across all transects. Second, emersion time per tile was matched with local water height records for the same 24 hours and extrapolated to the whole year to quantify the proportion of time each tile remained emersed throughout the year accounting for tidal cycles and storm swelling (between 0 and 1). We corrected for the datum by using the lowest emersion ratio value obtained with the temperature loggers as a benchmark (mean lower low water). Thirdly, we obtained shore height elevation per tile using a RTK GPS (Trimble Catalyst DA1) and extrapolated annual emersion time across the shore using a second polynomial regression (adjusted R^2^ = 0.95). To allow the assessment of heterogeneity effects under different levels of emersion stress, the resulting emersion rate was split into three categories (i.e., low, mid, high) with each containing the same number of stations (i.e., each station containing two tiles).

Supporting Information S3: Consumer occurrence

Due to their high mobility, consumer cover could have shown large variability across seasons and thus not been captured by mean consumer cover values. Despite this variability, however, mean consumer abundance and cover values were representative of consumer abundance and cover at any single time point, respectively (Figure S2).

**Figure S2. The correlation between mean consumer abundance and cover and consumer abundance and cover values, respectively.** Consumer cover values showed 0.759 and 0.75 correlation with mean consumer abundance and cover, respectively. The dashed line indicates the 1:1 correlation corresponding to mean abundance and cover values, respectively.

Supporting Information S4: Stability metrics used in the study

**Table S3. Stability-related metrics quantified in this study, their quantification, definition and interpretation.**

| Metric | Quantification | Definition | Interpretation |
| --- | --- | --- | --- |
| Temporal Stability (aggregate) | $\frac{\mu_{tot}}{\sigma_{tot}}$ | Mean value of an aggregate community of ecosystem property ($\mu_{tot}$) across time points, divided by itsstandard deviation ($\sigma_{tot}$) across time. In this study we used total community cover as the focal aggregate community property ^4^ | Measures the temporal stability of the entire community’s abundance, biomass, or, as in this study, cover. Higher values indicate more stable aggregate properties. Also known as aggregate or functional stability. |
| Population Stability | $\frac{\sum_{i,k} \mu_{i,k}}{\sum_{i,k} \sqrt{\upsilon_{ii,kk}}}$ | Sum of mean abundance, biomass or - as in this study -cover per species(*μ_ik_*) across time in community k, divided by the sum of the standard deviation per species (*𝜈_i_*_i_). ^1^. | Indicates the average temporal stability of individual species in a community. Higher population stability generally contributes to greater aggregate stability. |
| Species Asynchrony | $-\sum_{i} \left[ p_{i}r\left( A_{i},\sum_{j\neq i} A_{j} \right) \right]$ | Negative sum of correlations (r) between each species i and each of all the other species j≠i, weighted by species i’s relative cover (*p_i_*). ^2^ | Asynchronous species fluctuations reduce simultaneous declines in cover, thereby buffering community-level variability and enhancing aggregate stability. |
| Statistical Averaging | $\frac{\sum_{i} \sigma_{i}}{\sqrt{\sum_{i} \sigma_{i}^{2}}}$ | Sum of standard deviations of species cover divided by the square root of the sum of species variances.^3^ | Reflects the portfolio effect, where random fluctuations average out over time, stabilizing total community cover. Higher values indicate stronger averaging.^5^ |
| Temporal Compositional Stability | $\frac{{\sum_{ijk} 1-\beta}_{BCijk}}{n_{k}}$ | Average Bray–Curtis similarity (${1-\beta}_{BC}$) between consecutive dates i and j for each tile k, divided by the number of time points ($n_{k}$). ^4^ | Assesses temporal consistency in species composition. Higher values indicate more stable species assemblages over time, contributing to overall community stability. |
| Liang et al. 2021^1^; Blüthgen et al. 2016^2^; Donohue et al. 2016^3^; Hillebrand et al. 2018^4^; Doak et al. 1998^5^ | | | |

**Figure S3. Visual representation of each stability metric considered in this study.** While population stability, species asynchrony and statistical averaging showed direct contributions to temporal stability by helping (or not) maintain constant species cover, temporal compositional stability contributed by maintaining stable composition both in terms of species identity and species cover per taxa. All metrics are measured per tile, using all time points available for each tile.

Supporting Information S5: Sensitivity tests regarding the effect of time on community components and temporal stability

*Testing the effect of time on the averaged temporal values across community components*

During the experimental period, communities showed some development over time (Figure S4). Pielou evenness, native barnacles and consumers seemed to increase steadily over time, while the dominant barnacle *Austrominius modestus* and species richness appeared to decrease over time.


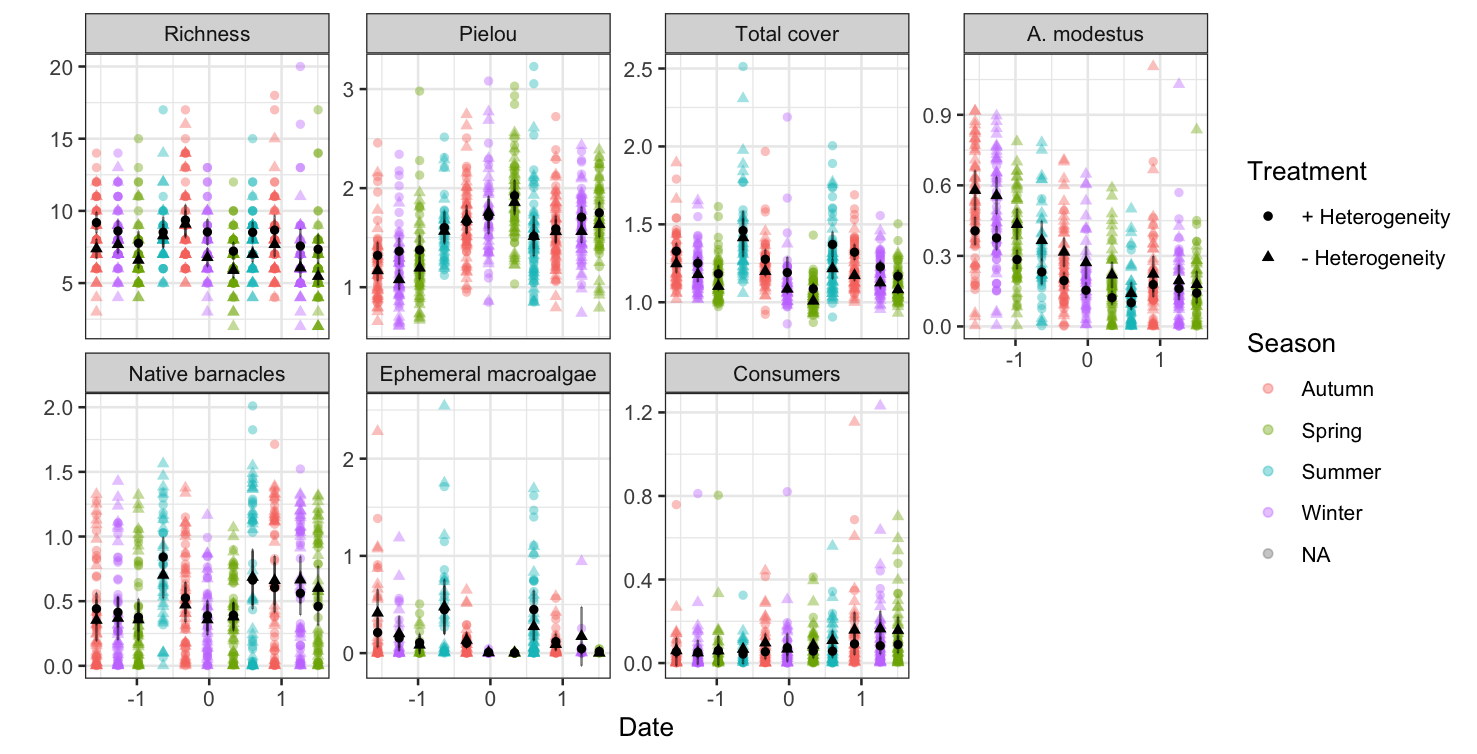


**Figure S4. Time series for species diversity and the cover of the main species groups.** In each panel, raw observations are shown, as well as the mean and standard error per date. Units for richness are number of species, for Pielou units are dimensionless, and for the reminder of panels units are a proportion representing species cover.

To evaluate whether temporal changes influenced the role of community components in driving stability mechanisms, we tested whether the effect of spatial heterogeneity across shore levels varied over time (Figure S5). To do so, we constructed a dataset comprising four subsets: (1) the full dataset including all years, (2) a dataset excluding the first year, (3) a dataset excluding the second year, and (4) a dataset excluding the last year. We found no significant differences in any of the models that included the three-way interaction between Emersion Ratio × Heterogeneity × Year, or in any other factor-level combinations. However, when examining the two-way interaction Heterogeneity × Year, we detected a significant effect for *A. modestus* during the first year, with mean cover higher than in subsequent years (t =-3.84; P<0.001). Differences across heterogeneity treatments among the various species groups and diversity metrics were maintained after accounting for the exclusion of data across various years.

Overall, we argue that spatial comparisons of temporal mean values effectively accounted for variability that over-rides patterns of change associated with ecological succession. In addition, the rapid development of the studied communities likely also contributed to dampen and thus minimise any potential influence of successional dynamics on the stability mechanisms evaluated in this study.


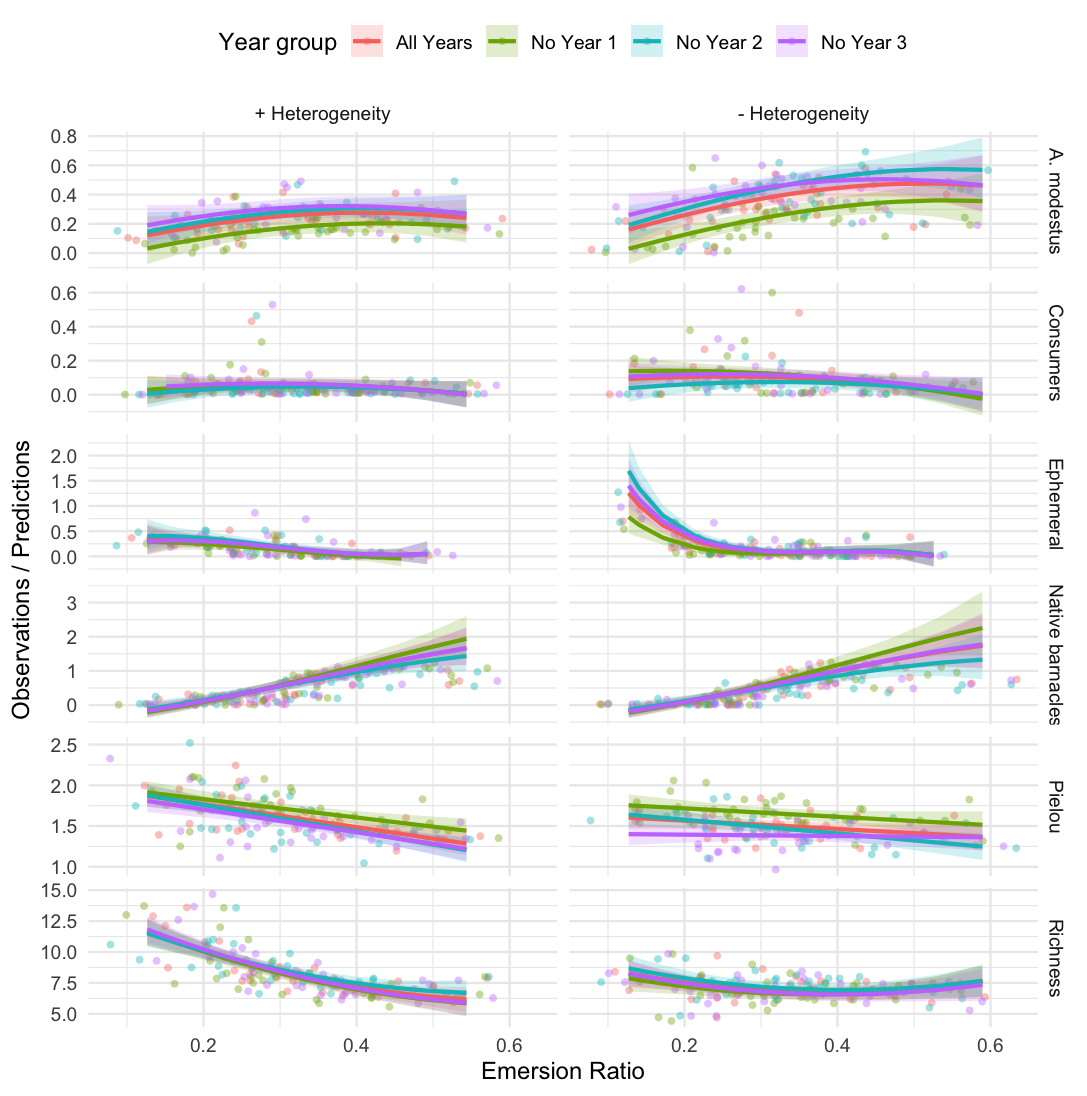


**Figure S5. Comparisons across community components after removing data for each year in the experiment.** In each row, we compare data for a given community component across heterogeneity treatments and emersion ratio values, considering the whole dataset (All Years), after excluding data for year 1 (No Year 1), year 2 (No Year 2) and year 3 (No Year 3).

*Testing the effect of year on temporal stability*

To ensure that any directional change in cover through time did not affect our estimates of temporal stability, we detrended time series data for total cover following methods in Craven et al. (2018). First, linear models were computed for each tile, using the time data for each tile. For example, for the first tile in the first transect, a linear model was build using the eleven time points available for that tile. The linear regression model included total cover as the response variable, and date as a predictor (in numerical format). Detrended temporal stability was then calculated using the following formula

Detrended temporal stability = $\frac{\mu_{tot}}{\sigma_{res}}$

where *μ_tot_* was the average total cover for all timepoints for a given tile and *σ_res_* was the standard deviation of the residuals generated with each linear model.

When then compared temporal stability to detrended temporal stability, which were closely correlated as indicated by Spearman’s correlation test (Figure S6).

**Figure S6. Comparison between temporal stability and detrended temporal stability.** The dashed line represents a 1:1 correlation between temporal stability and detrended temporal stability, and Spearman correlation (⍴) is quantified for each comparison.

Detrending temporal stability showed low sensitivity to year exclusion (Figure S7). After sequentially removing each year from the dataset, we found that after removing the first year detrended stability remained highly correlated (*ρ* = 0.92), which was similar to the correlation values obtained after removing year 2 or year 3 from the dataset.

**Figure S7. Detrended temporal stability comparisons after removing data for each year in the experiment.** In each panel, detrended temporal stability was re-calculated (i.e., modified temporal stability) to exclude year 1 (Stability minus year 1), year 2 (Stability minus year 2) and year 3 (Stability minus year 3). The dashed line indicates the 1:1 correlation between the modified temporal stability and temporal stability, and Spearman correlation (⍴) is quantified for each comparison.

We also performed sensitivity tests to check whether detrending temporal stability had any effects in the results of this study (Table S4). To do so, we used the same mixed-effects linear model used to test heterogeneity effects on temporal stability – presented in Figure 2 in the results section of this study. We observed very small changes in the model outputs, with no changes pertaining to heterogeneity effects.

**Table S4. Sensitivity test between mixed-effects linear models testing for heterogeneity effects and emersion rate effects on detrended and non-detrended temporal stability.** Differences in significance indicate cases where effects switched significance between models.

| Fixed effects | Estimate (SE) | | p-value | | Differences in significance |
| --- | --- | --- | --- | --- | --- |
|  | Not detrended | Detrended | Not detrended | Detrended |  |
| Heterogeneity | -0.41 (0.23) | -0.38 (0.22) | 0.079 | 0.100 |  |
| Emersion rate | -48.03 (21.40) | -36.77 (18.45) | 0.031 | 0.055 | * |
| Emersion rate^2^ | 404.13 (127.20) | 335.97 (109.74) | 0.003 | 0.004 |  |
| Emersion rate x Heterogeneity | 1.95 (1.34) | 1.85 (1.32) | 0.155 | 0.170 |  |

Supporting Information S6: Metamodel for the testing of heterogeneity effects on Temporal Stability

To build the Structural Equation Models, we established causal links across variables of interest (Figure S8) following preestablished cause-effect links in the literature for a) heterogeneity effects on species diversity and composition and b) species diversity and composition effects on temporal stability variables.

First, we hypothesised that heterogeneity would promote species richness and species evenness (Stein et al. 2014). In addition, heterogeneity would limit consumers (Griffin et al. 2009) and may also be linked with the abundance of other dominant species groups in the community (Hauser et al. 2006) such as *Austrominius modestus*, native barnacles and ephemeral macroalgae.

Second, links between species richness, species evennes, and dominant species groups (including consumers) with stability metrics were guided by multiple hypotheses:

- Species richness will increase species asynchrony and statistical averaging, as the more species occurring within a plot will lead to higher competition across species, and it will increase the chance that species respond differently to a given change in environmental conditions, or that species recruit at different given times (Schnabel et al. 2021).
- Species richness may decrease population stability, since the arrival of more species will lead to the establishment of less-adapted species that do not survive environmental fluctuations.
- Species richness may decrease compositional stability, since the more species we add to a community, the higher the chance that less-adapted (less stress-tolerant) species we add to the community, which decrease compositional stability (Sankaran & McNaughton 1999).
- Species evennes may decrease species asynchrony, since the decrease in dominant species will decrease competitive interactions within the community.
- Species evenness may increase statistical averaging, since the decrease in dominant species may release space for other species to colonise.
- Species evenness may decrease population stability, as the decrease of dominant well-adapted species may allow other rarer and less stress-tolerant species to colonise, which will be wiped out with environmental fluctuations.
- Species evennes may decrease compositional stability for similar reasons to decreased dominant species and species richness effects on compositional stability.
- Consumers may decrease ephemeral macroalgae, *A. modestus* and native barnacles (Menge et al. 2010). Consumers will also decrease competition within the community through competition release effects, thus decresing species asynchrony, and increase statistical averaging by releasing space and thus allow the recruitment of other species. Consumers may increase population stability and compositional stability by promoting the presence of species tolerant to grazing, which present traits that also make them more tolerant to stress (e.g., banracles). In addition, consumers may keep overall community cover low and keep the remaining organisms inside the pits where they can’t access. This keeps organisms inside refugia, where they will be less affected by disturbance and thus increase compositional stability and population stability.
- Dominant barnacles species (*A. modestus* and native barnacles) may increase species asynchrony by increasing competition within the community, reduce statistical averaging by reducing the availability of free space for new species to randomnly recruit, increase population stability and compositional stability by increasing the stability of the overall communtiy since these are stress-tolerant species (Lisner et al. 2024).
- Ephemeral macroalgae may decrease population stability and compositional stability since they are highly seasonal and stress-sensitive species. Given their wide fluctuations, they may positively contribute to species asynchrony and statistical averaging (Pihl et al. 1996).

In agreement with our hypotheses stated earlier in the study and previous evidence and theory (Liang et al. 2021), we hypothesised that heterogeneity may be linked to temporal stability, compositional stability, population stability, species asynchrony and statistical averaging. We further hypothesised that population stability, species asynchrony and statistical averaging would be linked to temporal stability, following established theory (Liang et al. 2021). We also included a link between compositional stability and temporal stability, since changes in the overall cover of the community, and the changes in the stability of dominant species will influence temporal stability. Laslty, and since population stability, species asynchrony and statistical averaging may affect the mean total cover as well as be the consequence of changes in dominant species and species diversity, we linked population stability, species asynchrony and statistical averaging to compositional stability.

**Figure S8. Hypothesised links used in the SEM analysis to test heterogeneity effects on temporal stability.** Metamodel indicating the hypothesised links between heterogeneity and community components and stability metrics, and the links between community components and stability metrics. Solid black lines indicate the hypothesised links among variables, with the pointed arrow indicating the causal direction in each link.

Supporting Information S7: Sensitivity Tests for the Randomised Block Design

We conducted sensitivity analyses to assess potential spatial confounding arising from the randomised block design—specifically, the concern that comparisons across heterogeneity treatments might be masked by local environmental variation across blocks or, in our case, stations. We re-ran the mixed-effects linear models using bootstrapped datasets that compared heterogeneity treatments either *only within* stations (i.e., within the same station or transect × tidal level combinations) or *only across* stations (i.e., only comparing heterogeneity treatments not found within the same station). Results were consistent across both approaches for all stability metrics, with significant effects retained in both cases (Figure S9). Only one coefficient (Emersion effects on Cover stability) was affected, becoming marginally significant, but not statistically different from the within-station model estimate. This indicates that our conclusions regarding environmental heterogeneity are robust to spatial structuring in the dataset.

Importantly, all stations were placed on relatively homogeneous exposed rock, avoiding localised features such as rock pools and macroalgal beds that could introduce environmental gradients. This, along with the close proximity of transects and selection of a shore with minimal across-transect variability, helped reduce unmeasured differences in local environmental variability. Additionally, we modelled transect and station as nested random effects, explicitly accounting for spatial variability and mitigating potential confounding.

While the design includes only one tile per rugosity level within each station—a recognised limitation—we maintain that the randomised block design remains suitable for this type of fully-crossed experiments (Hartley et al. 1953; Krzywinski et al. 2014). When paired with thoughtful experimental setup, site selection and appropriate random error structure, it effectively balances the need to control spatial noise while preserving the ability to detect treatment effects. Overall, our design, combined with multiple sensitivity checks and spatial controls, supports the conclusion that our results reflect true ecological patterns rather than artefacts of spatial structure.


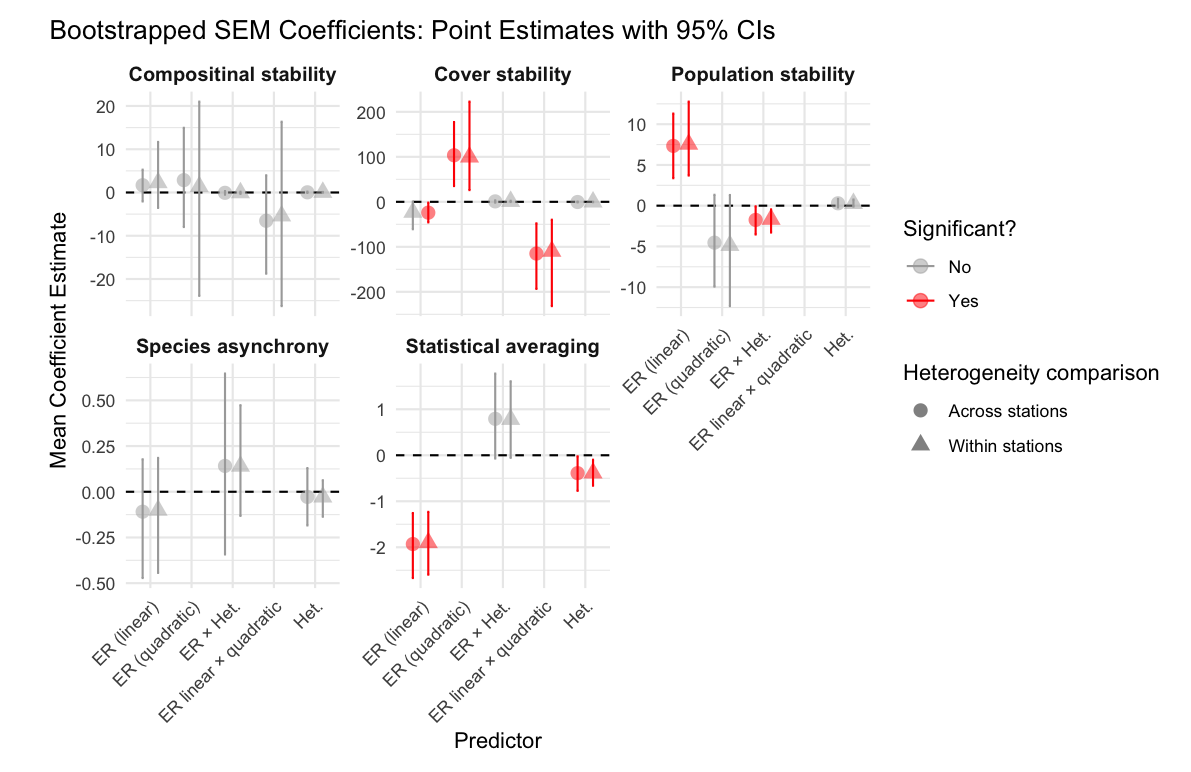


**Figure S9. Sensitivity analysis comparing bootstrapped linear model coefficients for all stability metrics evaluated *only within* stations (i.e., within transect × tidal level combinations) versus *only across* stations.** The plots show point estimates and 95% confidence intervals for all stability metrics. Significant effects are highlighted in red. Results demonstrate that key findings are robust across both comparisons, with only one coefficient (Emersion effects on Cover stability) becoming marginally significant. Abbreviations: ER = Emersion Ratio, Het. = Heterogeneity.

Supporting Information S8: Sensitivity tests for the Multigroup Piecewise SEM model

Excluding whelks and considering only limpets as consumers had minimal impact on the multigroup SEM results (Figure S10). The only notable changes were that *Austrominius modestus* was no longer significantly affected by emersion ratio in the mid and high shore, and limpets showed a significant effect on statistical averaging—though this did not cascade to compositional or cover stability. Whelks fulfil a distinct and complementary trophic role, as they feed primarily on barnacles and mussels rather than algae (Feare 1970; Fairweather & Underwood 1983). This difference may help explain the barnacle response to emersion in those zones. Additionally, grouping limpets and whelks under a general 'consumer' category supports conceptual consistency and facilitates cross-system generalisation, particularly when consumers differ taxonomically but share functional roles (Menge et al. 1986; Silliman et al. 2013). For these reasons, we retained both limpets and whelks in the analysis.


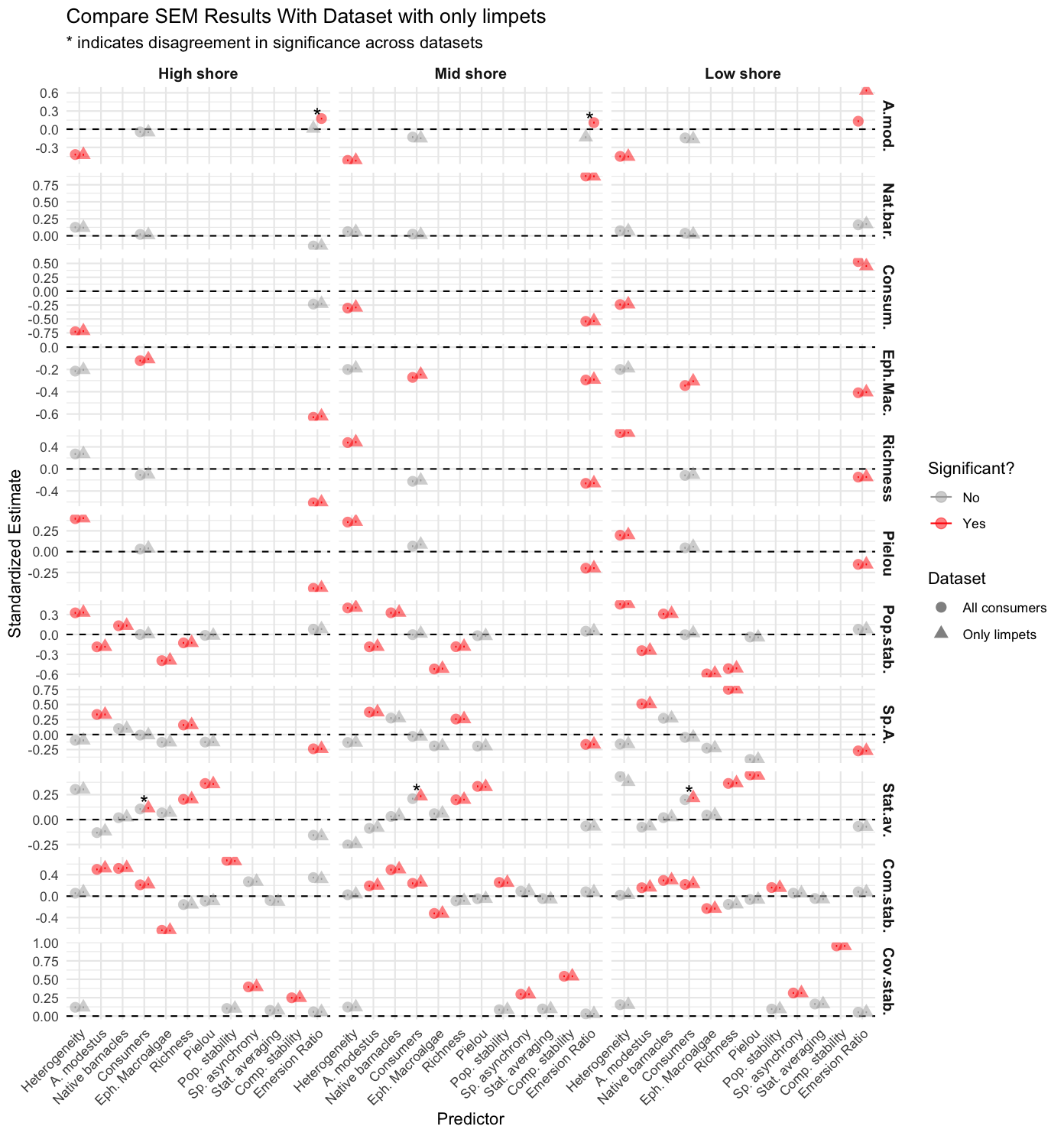


**Figure S10. Sensitivity tests comparing multigroup SEM results *with all consumers* vs. *with only limpets*.** Each plot shows the standardised coefficient for the multigroup SEM, which has no standard error or confidence interval as per the output of the function in the package piecewiseSEM. Cases where the significance of coefficients changed across the two datasets are indicated with an asterisk (*). Abbreviations: A. modestus / A.mod. = *Austrominius modestus*, Nat.bar = Native barnacles, Consum. = Consumers (all consumers in original dataset, only limpets in limpet dataset), Eph. Macroalgae / Eph.Mac. = Ephemeral macroalgae, Pop. stability / Pop.stab. = Population stability, Sp. Asynchrony / Sp.A. = Species asynchrony, Stat. averaging / Stat.av. = Statistical averaging, Comp. stability / Com.stab. = Compositional stability, Cov.stab. = Cover stability.

During our SEM analysis, we also considered removing two variables—Pielou’s evenness and Statistical Averaging—that did not directly or indirectly affect Cover Stability (Figure S11). While simplifying the model had little overall impact on our results, it eliminated a crucial link quantifying the negative effects of species richness on compositional stability. We believe that the Pielou index captured variability among moderately abundant species, whereas Statistical Averaging accounted for stochastic fluctuations in species abundance. Such context‐explanatory variables are vital in SEMs for disentangling complex, coexisting relationships (Grace, 2006; Shipley, 2000). Moreover, retaining these variables revealed three rarely reported connections—the link between heterogeneity and the Pielou index, and those between Pielou’s index and Richness with Statistical Averaging. Considering both their contextual explanatory value and the emergence of new links, we opted to retain the complete model.


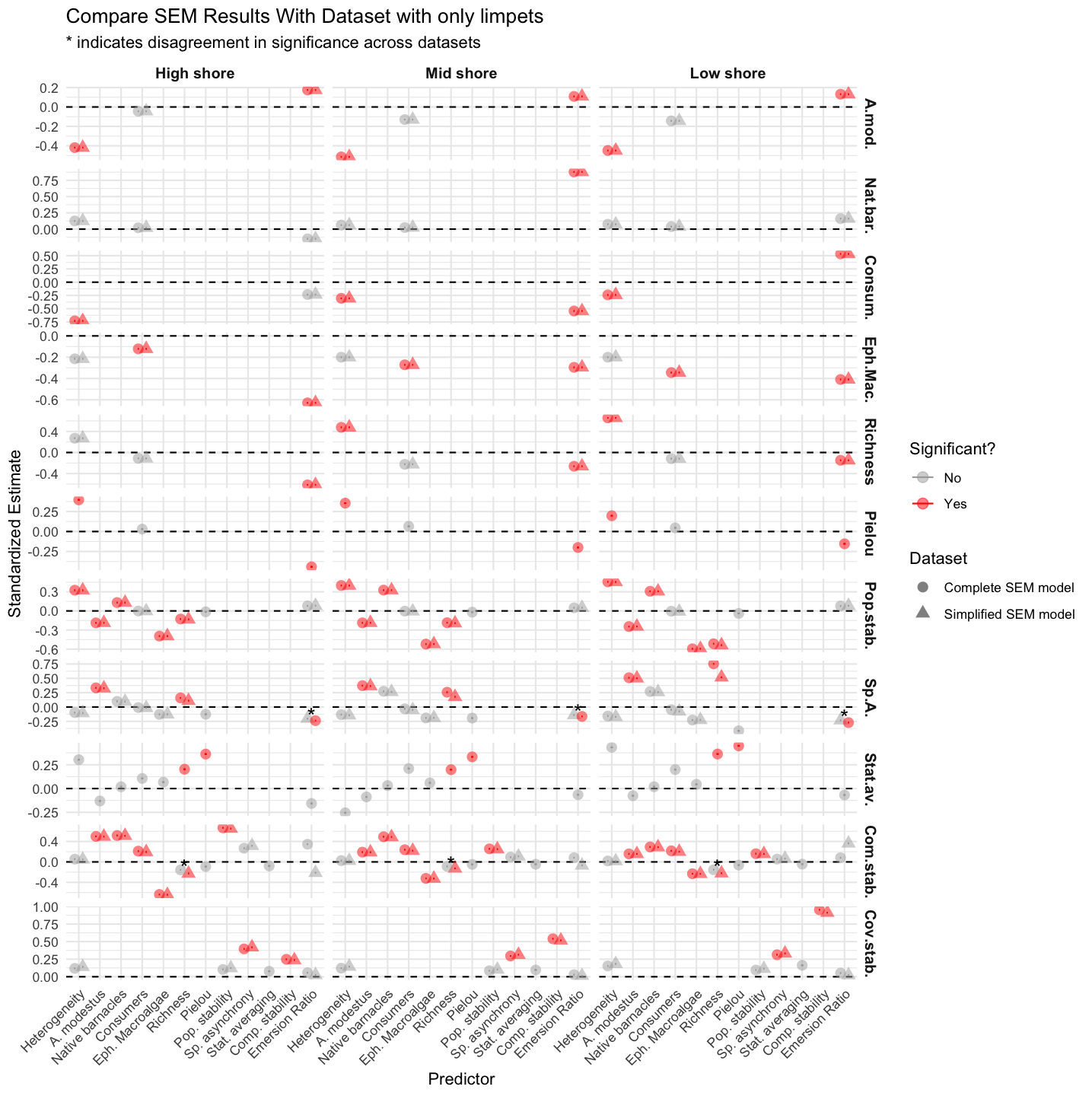


**Figure S11. Sensitivity tests comparing multigroup SEM results with all variables vs a simplified model obtained by removing two variables (Pielou and Statistical averaging).** Each plot shows the standardised coefficient for the multigroup SEM, which has no standard error or confidence interval as per the output of the function in the package piecewiseSEM. Cases where the significance of coefficients changed across the two datasets are indicated with an asterisk (*). Abbreviations: A. modestus / A.mod. = *Austrominius modestus*, Nat.bar = Native barnacles, Consum. = Consumers (all consumers in original dataset, only limpets in limpet dataset), Eph. Macroalgae / Eph.Mac. = Ephemeral macroalgae, Pop. stability / Pop.stab. = Population stability, Sp. Asynchrony / Sp.A. = Species asynchrony, Stat. averaging / Stat.av. = Statistical averaging, Comp. stability / Com.stab. = Compositional stability, Cov.stab. = Cover stability.

SI References

Blüthgen, N., Simons, N. K., Jung, K., Prati, D., Renner, S. C., Boch, S., ... & Gossner, M. M. (2016). Land use imperils plant and animal community stability through changes in asynchrony rather than diversity. *Nature Communications*, *7*(1), 10697.

Craven, D., Eisenhauer, N., Pearse, W. D., Hautier, Y., Isbell, F., Roscher, C., ... & Manning, P. (2018). Multiple facets of biodiversity drive the diversity–stability relationship. *Nature ecology & evolution*, *2*(10), 1579-1587.

Doak, D. F., Bigger, D., Harding, E. K., Marvier, M. A., O'malley, R. E., & Thomson, D. (1998). The statistical inevitability of stability-diversity relationships in community ecology. *The American Naturalist*, *151*(3), 264-276.

Donohue, I., Hillebrand, H., Montoya, J. M., Petchey, O. L., Pimm, S. L., Fowler, M. S., ... & Yang, Q. (2016). Navigating the complexity of ecological stability. *Ecology letters*, *19*(9), 1172-1185.

Fairchild, T. (2019). Small scale substrate complexity enhances multiple facets of biodiversity across environmental contexts. In thesis: The Causes and Consequences of Variation in Different Dimensions of Biodiversity on Rocky Shores pp. 44-84.

Fairweather, P. G., & Underwood, A. J. (1983). The apparent diet of predators and biases due to different handling times of their prey. *Oecologia*, *56*, 169-179.

Feare, C. J. (1970). Aspects of the ecology of an exposed shore population of dogwhelks Nucella lapillus (L.). *Oecologia*, *5*, 1-18.

Grace, J. B. (2006). *Structural equation modeling and natural systems*. Cambridge University Press.

Griffin, J. N., Jenkins, S. R., Gamfeldt, L., Jones, D., Hawkins, S. J., & Thompson, R. C. (2009). Spatial heterogeneity increases the importance of species richness for an ecosystem process. *Oikos*, *118*(9), 1335-1342.

Hartley, H. O., Shrikhande, S. S., & Taylor, W. B. (1953). A note on incomplete block designs with row balance. *The Annals of Mathematical Statistics*, 123-126.

Hauser, A., Attrill, M. J., & Cotton, P. A. (2006). Effects of habitat complexity on the diversity and abundance of macrofauna colonising artificial kelp holdfasts. *Marine Ecology Progress Series*, *325*, 93-100.

Hillebrand, H., Langenheder, S., Lebret, K., Lindström, E., Östman, Ö., & Striebel, M. (2018). Decomposing multiple dimensions of stability in global change experiments. *Ecology letters*, *21*(1), 21-30.

Krzywinski, M., Altman, N. & Blainey, P. Nested designs. *Nat Methods* **11**, 977–978 (2014). https://doi.org/10.1038/nmeth.3137

Liang, M., Liang, C., Hautier, Y., Wilcox, K. R., & Wang, S. (2021). Grazing‐induced biodiversity loss impairs grassland ecosystem stability at multiple scales. *Ecology Letters*, *24*(10), 2054-2064.

Lisner, A., Segrestin, J., Konečná, M., Blažek, P., Janíková, E., Applová, M., ... & Lepš, J. (2024). Why are plant communities stable? Disentangling the role of dominance, asynchrony and averaging effect following realistic species loss scenario. *Journal of Ecology*.

Menge, B. A., Lubchenco, J., Ashkenas, L. R., & Ramsey, F. (1986). Experimental separation of effects of consumers on sessile prey in the low zone of a rocky shore in the Bay of Panama: direct and indirect consequences of food web complexity. *Journal of Experimental Marine Biology and Ecology*, *100*(1-3), 225-269.

Menge, B. A., Foley, M. M., Pamplin, J., Murphy, G., & Pennington, C. (2010). Supply-side ecology, barnacle recruitment, and rocky intertidal community dynamics: Do settlement surface and limpet disturbance matter?. *Journal of Experimental Marine Biology and Ecology*, *392*(1-2), 160-175.

Pihl, L., Magnusson, G., Isaksson, I., & Wallentinus, I. (1996). Distribution and growth dynamics of ephemeral macroalgae in shallow bays on the Swedish west coast. *Journal of Sea Research*, *35*(1-3), 169-180.

Sankaran, M., & Mcnaughton, S. J. (1999). Determinants of biodiversity regulate compositional stability of communities. *Nature*, *401*(6754), 691-693.

Schnabel, F., Liu, X., Kunz, M., Barry, K. E., Bongers, F. J., Bruelheide, H., ... & Wirth, C. (2021). Species richness stabilizes productivity via asynchrony and drought-tolerance diversity in a large-scale tree biodiversity experiment. *Science advances*, *7*(51), eabk1643.

Schreider, M. J., Glasby, T. M., & Underwood, A. J. (2003). Effects of height on the shore and complexity of habitat on abundances of amphipods on rocky shores in New South Wales, Australia. *Journal of experimental marine biology and ecology*, *293*(1), 57-71.

Shipley, B. (2000). A new inferential test for path models based on directed acyclic graphs. *Structural Equation Modeling*, *7*(2), 206-218.

Silliman, B. R., McCoy, M. W., Angelini, C., Holt, R. D., Griffin, J. N., & van de Koppel, J. (2013). Consumer fronts, global change, and runaway collapse in ecosystems. *Annual Review of Ecology, Evolution, and Systematics*, *44*(1), 503-538.

Sousa, W. P. (1985). Disturbance and patch dynamics on rocky intertidal shores. *The ecology of natural disturbance and patch dynamics*, 101-124.

Stein, A., Gerstner, K., & Kreft, H. (2014). Environmental heterogeneity as a universal driver of species richness across taxa, biomes and spatial scales. *Ecology letters*, *17*(7), 866-880.
